# Device-Free Multi-Location Human Activity Recognition Using Deep Complex Network

**DOI:** 10.3390/s22166178

**Published:** 2022-08-18

**Authors:** Xue Ding, Chunlei Hu, Weiliang Xie, Yi Zhong, Jianfei Yang, Ting Jiang

**Affiliations:** 1 Mobile and Terminal Technology Research Department, China Telecom Research Institute, Beijing 102209, China; 2 School of Information and Electronics, Beijing Institute of Technology, Beijing 100081, China; 3 School of Electrical and Electronics Engineering, Nanyang Technological University, Singapore 639798, Singapore; 4 School of Information and Communication Engineering, Beijing University of Posts and Telecommunications, Beijing 100876, China

**Keywords:** human activity recognition, Wi-Fi sensing, multi-location, deep complex network

## Abstract

Wi-Fi-based human activity recognition has attracted broad attention for its advantages, which include being device-free, privacy-protected, unaffected by light, etc. Owing to the development of artificial intelligence techniques, existing methods have made great improvements in sensing accuracy. However, the performance of multi-location recognition is still a challenging issue. According to the principle of wireless sensing, wireless signals that characterize activity are also seriously affected by location variations. Existing solutions depend on adequate data samples at different locations, which are labor-intensive. To solve the above concerns, we present an amplitude- and phase-enhanced deep complex network (AP-DCN)-based multi-location human activity recognition method, which can fully utilize the amplitude and phase information simultaneously so as to mine more abundant information from limited data samples. Furthermore, considering the unbalanced sample number at different locations, we propose a perception method based on the deep complex network-transfer learning (DCN-TL) structure, which effectively realizes knowledge sharing among various locations. To fully evaluate the performance of the proposed method, comprehensive experiments have been carried out with a dataset collected in an office environment with 24 locations and five activities. The experimental results illustrate that the approaches can achieve 96.85% and 94.02% recognition accuracy, respectively.

## 1. Introduction

Human Activity Recognition (HAR) has been considered as an indispensable technology in many Human-Computer Interaction (HCI) applications, such as smart homes, health-care services, security surveillance, entertainment, etc [1,2]. Both the device-based and device-free sensing approaches attract widespread attention [3,4,5,6]. Owing to their superiority involving sensing accuracy and robustness, sensor-based [7,8] and camera-based [9,10] HAR methods have been widely used in various fields. However, these techniques experience varied limitations in some applications. Specifically, sensor-based methods require the users to equip themselves with additional devices, which is inconvenient. Although the camera-based technique is successfully applied to various scenarios, it is restricted to well-lit conditions and fails to work in a non-line-of-sight (NLOS) scene. More critically, it raises privacy concerns.

Recently, device-free sensing technology based on wireless signals has been widely studied owing to its capacity to overcome the above defects effectively [11,12]. Since only radio frequency (RF) signals are utilized, it naturally has the strengths of working in darkness and NLOS circumstances and protecting users’ privacy in the meantime. Compared with the other wireless signals, such as Frequency Modulated Continuous Wave (FMCW) [13,14], millimeter-wave (MMW) [15,16], and Ultra-Wide Band (UWB) [17,18], Wi-Fi has an overwhelming advantage due to its ubiquity in daily life. Leveraging commercial off-the-shelf (COTS) devices, Wi-Fi-based human activity recognition obviates the need for additional specialized hardware. Consequently, study of the Wi-Fi-based HAR technique has proliferated rapidly over the past decade [19,20,21,22].

Although Wi-Fi-based HAR approaches have made significant achievements at a fixed location, it is still challenging in multi-location sensing. When it comes to intelligent control in smart homes, it will be seriously inconvenient for users if they can only control the smart devices at a specified location in a room. Moreover, a target that can be detected at one position but fails to be identified at other positions is not desired. Therefore, multi-location sensing is one of the most essential capabilities for the HAR system. According to the principle of RF signal propagation, when encountering an obstacle, the signal will be reflected, refracted, and scattered, leading to the superposition of multipath [23,24]. Therefore, both the activity and its location affect signal transmission to a certain extent. Consequently, even for the same human activity, different locations would result in signals having different patterns, which will lead to a serious decline in multi-location sensing accuracy. Furthermore, owing to the rapid development of the deep learning technique, the performance of human activity recognition has been effectively improved [25]. However, these methods usually rely on abundant labeled or unlabeled samples, which have never been easily accessible for labor-intensive and time-consuming. In addition, it is quite difficult to obtain large amounts of data for all locations. Therefore, a multi-location human activity recognition method using small-scale data needs to be explored.

Literature [26] is the state-of-the-art multi-location human activity recognition method. An activity decomposition network (ActNet) is proposed to decompose the training samples into activity features and location features. In addition, data from different locations are assembled for training to mitigate the data limitation issue. The performance is evaluated at 24 sampling locations in the perceptual range. Using only 10 training samples for each position can achieve promising recognition accuracy.

Existing Wi-Fi-based sensing approaches for human activity recognition are mostly dependent on the amplitude of Channel State Information (CSI) because phase information contains certain errors caused by the hardware and software of the transceiver, including Sampling Time Offsets (STO) and Carrier Frequency Offsets (CFO) [27,28]. The proposed phase offsets removal method and phase difference make it possible to utilize phase information in Wi-Fi-based sensing [29,30]. However, it inevitably loses some useful information. Despite both amplitude and phase providing a wealth of activity-related information, only very few studies have used them simultaneously. In multi-location HAR, although the same activity conducted in different locations will lead to different signal delays at the receiver, the time delay generated by the same action may change with a certain rule, which is unrelated to the locations and will be reflected in the phase information. Therefore, leveraging the amplitude and phase information of CSI effectively at the same time can extract the feature representation that is more related to the activity. Furthermore, since more abundant features can be obtained, the sample size can be reduced to some extent. Although deep learning-based HAR methods emerge due to the key merit of automatically learning representative features, the amplitude and phase information are usually applied as the input of the neural network separately.

In this paper, inspired by the Deep Complex Network (DCN) [31,32] which is designed to extract meaningful information from the real and imaginary parts of complex numbers, we propose a multi-location human activity recognition system based on the Deep Complex Network. Firstly, we propose a multi-location human activity identification method based on Amplitude and Phase enhanced Deep Complex Network (AP-DCN), which can make efficient use of amplitude and phase information. Specifically, complex Convolutional Neural Network (CNN), complex Batch Normalization (BN), and the complex ReLU activation function are used for feature extraction. Softmax is used for activity classification. Under the condition of limited data samples, high accuracy multi-location human activity recognition is realized. Moreover, considering the imbalanced number of samples in different positions and the more restricted number of training samples in some positions, the transfer learning method is used to realize the sharing of human activity characteristics in distinct locations. A novel human activity sensing method based on Deep Complex Network-Transfer Learning (DCN-TL) is proposed. The model is trained with sufficient activity samples from source domain locations to learn the common features of the source domain and target domain, as well as the specific characteristics of the source domain. Then, the model is fine-tuned with a small number of samples from target domain locations to learn the specific characteristics of the target domain. Thereby, in the case of unbalanced samples at different locations, multi-location human activity recognition can be achieved.

The main contributions of this paper can be summarized as follows:

- First, the effects of amplitude and phase information on Wi-Fi-based human activity recognition are analyzed. In order to make full use of the information in CSI, the AP-DCN-based recognition method is designed to improve the recognition accuracy of multi-location sensing.
- Second, in order to alleviate the problem of unbalanced samples at different locations, the DCN-TL-based human activity recognition method is proposed to reduce the dependence of the perception method on the number of activity samples at a specific location.
- Third, comprehensive experiments are conducted to evaluate the performance of the proposed AP-DCN-based and DCN-TL-based multi-location sensing methods. Experimental results demonstrate that the proposed approach can achieve satisfactory multi-location human activity recognition accuracy with very few samples.

The remainder of this article is organized as follows. In Section 2, the preliminaries of Wi-Fi sensing are introduced. In Section 3 provides an overview of the proposed system and a detailed description of the AP-DCN-based and DCN-TL-based multi-location human activity recognition methods. In Section 4, the experiment setup and performance evaluation are elaborated. Our conclusions are presented in Section 5.

## 2. Preliminaries

In this section, the measured signal is analyzed to verify the multi-location issue mentioned above. Firstly, the signal metric leveraged in Wi-Fi-based HAR is introduced. Then, the influence of location variations on Wi-Fi signals is investigated to reveal the encountered challenges. More importantly, both amplitude and phase information are presented.

### 2.1. Channel State Information

Wi-Fi-based HAR leverages the impact of human movements on the propagation of the wireless signal for sensing. In a Multiple Input Multiple Output (MIMO) and Orthogonal Frequency Division Multiplexing (OFDM) wireless communication system, this process can be described by the fine-grained CSI, which represents the state of the communication link between the transmitter (TX) and the receiver (RX).

Letting y and x denote the received signal and transmitted signal, the relation between them can be modeled as:

(1) y=Hx+n

where n is the noise vector and H is the CSI channel matrix which is made up of complex numbers, namely $H=H_{R}+iH_{I}$. For the *s*-th subcarrier between the *i*-th transmitting antenna and the *j*-th receiving antenna, it is given by

(2) $$H_{ij}^{s}=∥H_{ij}^{s}∥e^{j∠H_{ij}^{s}},s\in \left[1,N_{s}\right],i\in \left[1,N_{t}\right],j\in \left[1,N_{r}\right]$$

where $∥H_{ij}^{s}∥$ and $∠H_{ij}^{s}$ denote amplitude and phase, respectively. $N_{t}$ and $N_{r}$ stand for the number of antennas at the TX and RX. And *i* and *j* are the indices of TX and RX antennas. $N_{s}$ is the number of subcarriers for each pair of transceiver antenna.

### 2.2. Problem Analysis

**Figure 1:**
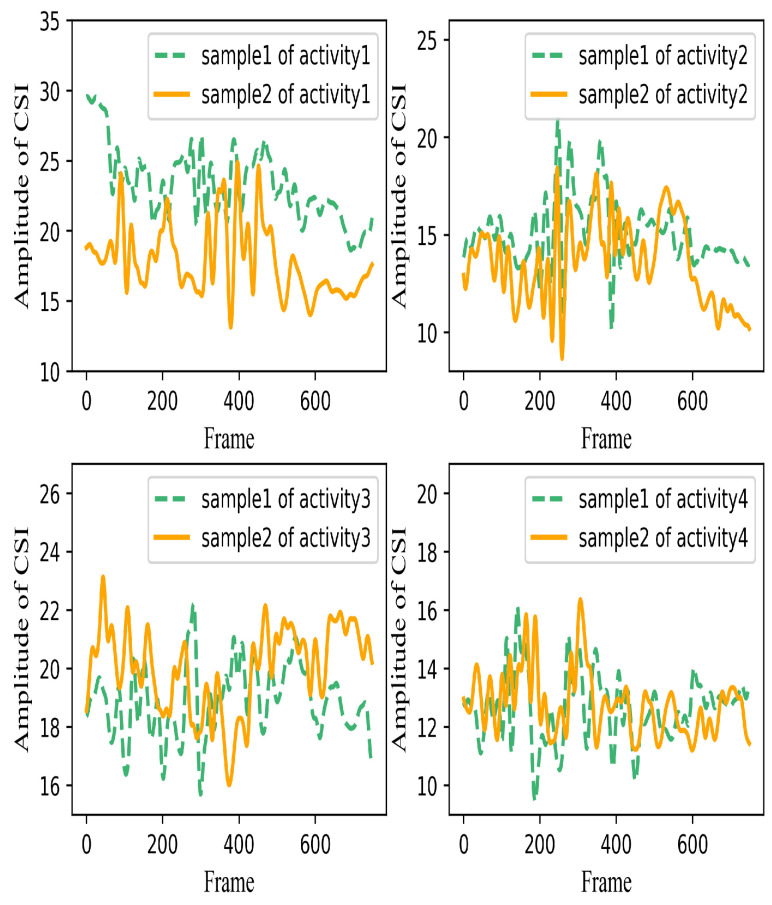
CSI amplitude of four different activities at the same location.

**Figure 2:**
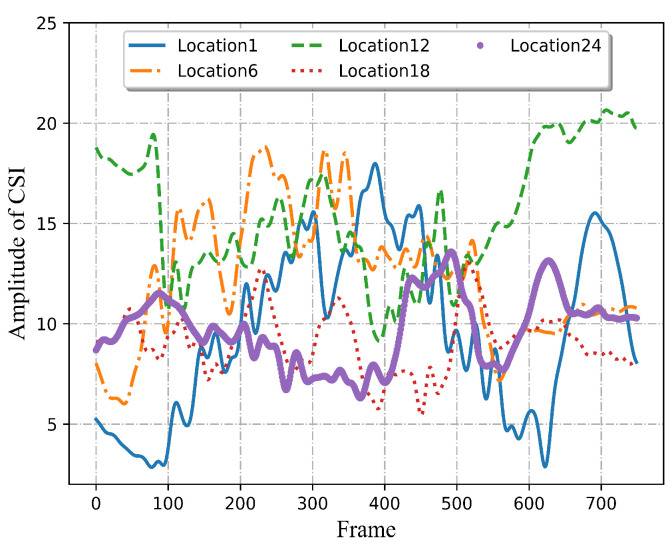
CSI amplitude of the same activity at five different locations.

To demonstrate the challenge of multi-location HAR using Wi-Fi signals, the CSI amplitudes of the activities at the same and distinct locations are analyzed and presented in Figure 1 and Figure 2. The horizontal axis represents the frame length, and the ordinate indicates the amplitude of CSI. The dataset will be presented in more detail in Section 4. As demonstrated in Figure 1, each subgraph depicts a kind of activity. The two curves in each subgraph represent two samples of the same activity. The measured signals for the same action at a fixed location seem to have similar waveforms. In the left figures of Figure 1, the variation trends of the two samples are different to some extent, which is because there are slight differences in the amplitude, speed, and starting position of each movement performed by the volunteers, resulting in differences among different samples of the same activity. Therefore, in the process of activity recognition, it is necessary to extract similar feature patterns between different samples of the same action that represent the changing trend of the activity itself as much as possible. Furthermore, it can be observed that diverse human activities will generate different characteristic patterns in the received signals at the same location. These are the foundation of wireless sensing. However, the measured signals for the same activity possess varying CSI amplitudes at different locations. As illustrated in Figure 2, five curves correspond to the same activity at five different locations. As can be seen, although it is relatively easy to identify the types of human activities by interpreting the CSI patterns at a single location, it may not be possible to ensure good classification accuracy for multi-location sensing.

**Figure 3:**
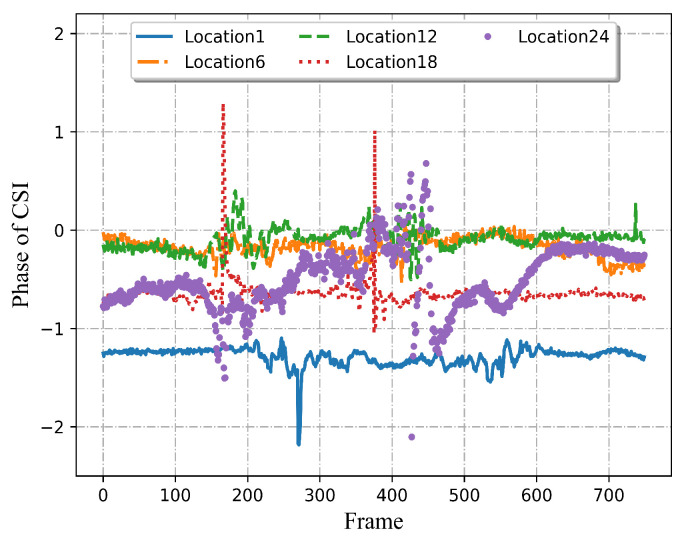
CSI phase of the same activity at five different locations.

In addition to the amplitude of CSI, the phase of the activity is also analyzed and shown in Figure 3. As can be seen, the phase can also reflect the characteristics of the activity. Therefore, both amplitude and phase should be utilized effectively. According to the above observation, just in terms of amplitude and phase, apart from the types of activities, the location variations can also obviously affect signal transmission. Therefore, both kinds of information should be strongly integrated, which can provide more accurate representative features so as to achieve multi-location sensing with limited data.

## 3. System Model

### 3.1. Overview

**Figure 4:**
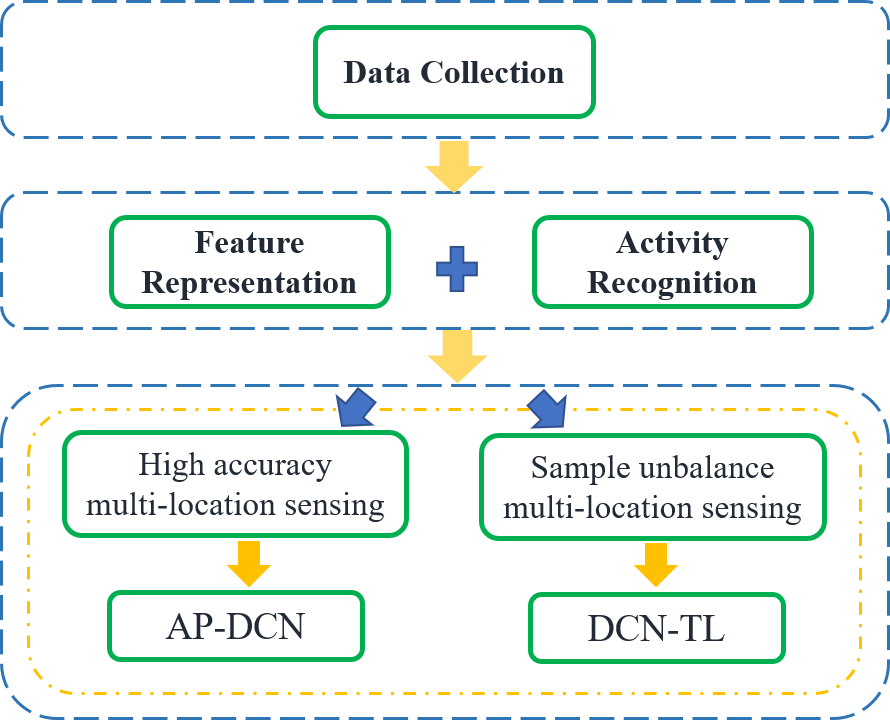
The framework of multi-location human activity recognition system.

In this part, the framework of the multi-location human activity recognition system is introduced, as shown in Figure 4. First of all, the Wi-Fi communication system is set up to collect CSI data in the Wi-Fi environment. The details will be described in Section 4. Then, feature extraction is carried out on CSI samples that are affected by human activities, and activity categories are distinguished according to the differences in features to realize human activity recognition.

To meet the requirement of high accuracy multi-location human activity recognition, a sensing method based on AP-DCN was proposed. High-accuracy multi-location sensing depends on adequate activity data from various positions. When data samples are restricted, sufficient activity information should be mined from the limited data. According to the above analysis, both the amplitude and phase of the Wi-Fi signal carry information related to human activities. Compared with the real-valued deep learning method based on the single amplitude or phase, the deep complex network simulates complex space computation through real-number space computation and can extract richer feature information. AP-DCN is designed to fully mine human activity information in amplitude and phase of CSI by using the complex convolution operation.

In some application scenarios, besides the lack of data samples, there is also the problem of unbalanced sample numbers provided at different positions. Therefore, a multi-location human activity perception method based on transfer learning named DCN-TL is proposed to transfer the common features of human activities learned from some locations with sufficient activity data to other locations with insufficient data so as to alleviate the impact of unbalanced data samples and limited sample number.

### 3.2. Ap-Dcn

#### 3.2.1. Network Architecture of AP-DCN

**Figure 5:**
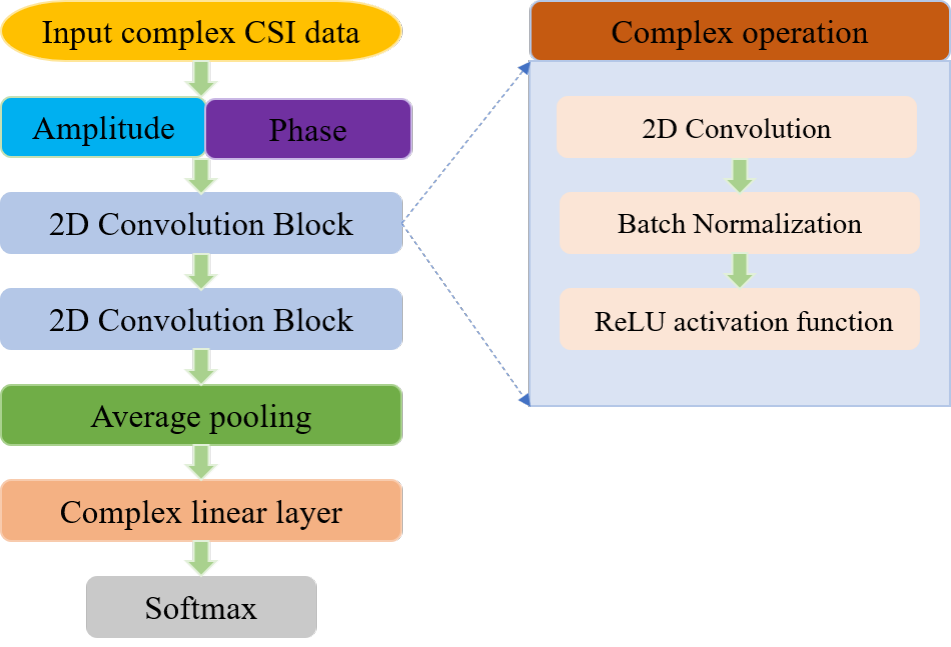
The architecture of the AP-DCN.

In this section, AP-DCN, which makes full use of the amplitude and phase information of CSI for multi-location human activity recognition, is designed. The architecture of the proposed AP-DCN is shown in Figure 5. With CSI as the input, in order to efficiently guide the network to learn meaning information, the calculated amplitude and phase are input to the backbone network as the real and imaginary parts of the new complex matrix, respectively.

The network consists of two complex convolution blocks, each of which contains a two-dimensional complex convolution layer, a complex batch normalization layer, and a complex activation function layer. When it comes to the number of network layers, theoretically, the more layers, the more effectively features can be extracted. However, in our scenario, the data samples are limited, and too many layers will easily lead to overfitting of the model. In addition, considering the complexity of the network in general, we designed the network using two complex convolutional blocks. Specifically, the two complex convolutional layers use 32 and 16 complex convolutional kernels, respectively. The kernel size is 3 × 3. Batch normalization layers correspond to 32 and 16 channels, respectively. Rectified Linear Unit (ReLU) is used as the activation function of the network. In order to reduce the number of model parameters and alleviate the over-fitting problem to some extent, the adaptive average pooling is applied to the real part and the imaginary part, and the size of the output feature map is 1 × 1. Subsequently, a complex linear layer follows, which is equivalent to the full connection layer of a real-valued neural network. The input size of the linear layer is 16, and the output size is 5, corresponding to five activity categories. Finally, since human activity recognition is defined as a classification problem, a softmax layer is connected to the end of the network to predict the category of the activity. The details are presented in the following.

#### 3.2.2. Network Layer of AP-DCN

For the CSI channel matrix $H=H_{R}+iH_{I}$, $H_{R}\in R,H_{I}\in R$, where each element is a complex number. The amplitude and the phase can be expressed as:

(3) $$A=∥H∥=\sqrt{H_{R}^{2}+H_{I}^{2}}$$

(4) $$P=∠H=\arctan\left(H_{I}/H_{R}\right)$$

**Figure 6:**
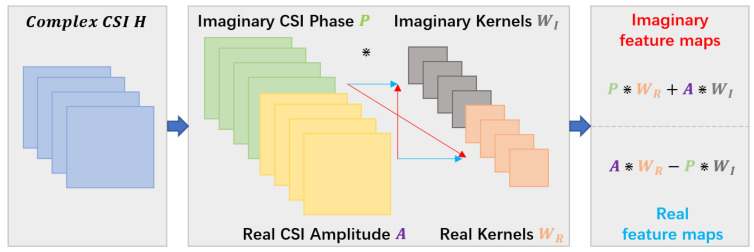
Complex convolution operation.

As described in the literature [31], to perform the two-dimensional convolution operations in the complex domain, complex filter matrix (complex convolution kernel) $W=W_{R}+iW_{I}$, where $W_{R}$ and $W_{I}$ are real matrices, is to be convolved by a complex matrix C=A+iP. The calculation process of complex convolution can be expressed as:

(5) $$\begin{array}{cc} W*C & =\left(W_{R}+iW_{I}\right)*\left(A+iP\right)\\ \; & =\left(W_{R}*A-W_{I}*P\right)+i\left(W_{I}*A+W_{R}*P\right) \end{array}$$

where * represents the convolution operation. The real and imaginary parts of the convolution operation can be expressed in matrix notation as follows:

(6) $$\left[\begin{array}{c} ℜ\left(W*C\right)\\ ℑ\left(W*C\right) \end{array}\right]=\left[\begin{array}{cc} W_{R} & -W_{I}\\ W_{I} & W_{R} \end{array}\right]*\left[\begin{array}{c} A\\ P \end{array}\right]$$

where *ℜ* and *ℑ* denote taking the real and imaginary parts of a complex number, respectively. The complex convolution operation is demonstrated in Figure 6.

To ensure the same distribution of the neural network input of each layer in the training process, and to effectively avoid the issue of the training gradient disappearing, complex batch normalization is used for transforming the input value of each layer to standard normal distribution with an average of 0 and variance of 1, so as to accelerate convergence speed of the deep model. Taking the input $x=\left\{x_{1},x_{2},\ldots ,x_{m}\right\}$ as an example, the output of the complex batch normalization layer can be obtained via the following process:

(7) $$\tilde{x}={\left(V\right)}^{-\frac{1}{2}}\left(x-E\left[x\right]\right)$$

where the expectation E is calculated as follows:

(8) $$E\left(x\right)=\left[\begin{array}{c} E\left(ℜ\left(x\right)\right)\\ E\left(ℑ\left(x\right)\right) \end{array}\right]=\left[\begin{array}{c} \frac{1}{m}\sum_{i=1}^{m}ℜ\left(x_{i}\right)\\ \frac{1}{m}\sum_{i=1}^{m}ℑ\left(x_{i}\right) \end{array}\right]$$

The covariance matrix *V* is

(9) $$\begin{array}{cc} V= & \left(\begin{array}{cc} V_{rr} & V_{ri}\\ V_{ir} & V_{ii} \end{array}\right)\\ = & \left(\begin{array}{cc} Cov\left(ℜ\left\{x\right\},ℜ\left\{x\right\}\right) & Cov\left(ℜ\left\{x\right\},ℑ\left\{x\right\}\right)\\ Cov\left(ℑ\left\{x\right\},ℜ\left\{x\right\}\right) & Cov\left(ℑ\left\{x\right\},ℑ\left\{x\right\}\right) \end{array}\right) \end{array}$$

where Cov implies the covariance calculation. Take $Cov\left(ℑ\left(x\right),ℜ\left(x\right)\right)$ as an example

(10) $$Cov\left(ℑ\left(x\right),ℜ\left(x\right)\right)=\frac{\sum_{i=1}^{m}\left(ℑ\left(x_{i}\right)-E\left(ℑ\left(x_{i}\right)\right)\right)\left(ℜ\left(x_{i}\right)-E\left(ℜ\left(x_{i}\right)\right)\right)}{m}$$

In order to maintain the original feature distribution, the scale transformation and translation transformation follow the calculation process in reference [31].

The complex ReLU (CReLU) activation function is applied on both the real and the imaginary part of a neuron. For a complex input *z*, it is given by

(11) CReLUz=ReLUℜz+iReLUℑz

The complex linear layer is computed similarly to the complex convolution operation by replacing the convolution operation with the multiplication operation. Then, the module value of complex output *z* of the complex linear layer is calculated to obtain:

(12) $$z^{\prime }=\sqrt{{\left(ℜ\left(z\right)\right)}^{2}+{\left(ℑ\left(z\right)\right)}^{2}}$$

In the training phase, cross-entropy loss is employed. Letting *L* denote a real-value loss function, the back-propagation (BP) can be written as:

(13) $$\begin{array}{cc} \nabla_{L}\left(H\right)= & \frac{\partial L}{\partial H}=\frac{\partial L}{\partial H_{R}}+i\frac{\partial L}{\partial H_{I}}=\frac{\partial L}{\partial ℜ\left(H\right)}+i\frac{\partial L}{\partial ℑ\left(H\right)}\\ = & ℜ\left(\nabla_{L}\left(H\right)\right)+iℑ\left(\nabla_{L}\left(H\right)\right) \end{array}$$

(14) $$\begin{array}{cc} \nabla_{L}\left(W\right)= & \frac{\partial L}{\partial W}=\frac{\partial L}{\partial W_{R}}+i\frac{\partial L}{\partial W_{I}}\\ = & ℜ\left(\nabla_{L}\left(H\right)\right)\left(\frac{\partial H_{R}}{\partial W_{R}}+\frac{\partial H_{R}}{\partial W_{I}}\right)\\ + & ℑ\left(\nabla_{L}\left(H\right)\right)\left(\frac{\partial H_{I}}{\partial W_{R}}+\frac{\partial H_{I}}{\partial W_{I}}\right) \end{array}$$

The loss function is minimized with Adam [33] to optimize the network parameters. The exponential decay rate ρ1 and ρ2 are empirically set as 0.9 and 0.999, and the learning rate is set as 0.001. ReduceLROnPlateau learning rate policy is utilized. The learning rate will be reduced by half when there is no improvement in the training loss over eight epochs. The total epoch is set up as 50.

### 3.3. Dcn-Tl

#### 3.3.1. Human Activity Recognition Method Based on DCN-TL

The process of the DCN-TL-based sensing method mainly consists of feature representation and recognition, and model fine-tuning based on transfer learning. A feature representation and classification recognition method based on a one-dimensional complex convolutional network was proposed to extract features and predict categories of human activities. The network is trained through the human activity samples with sufficient data in the source domain to obtain the pre-trained model. Then, a small number of target domain samples are used to update the pre-trained model by the transfer learning method. In practical application scenarios, the model pre-training and the model update are completed offline. After the optimal parameters are obtained, the activity prediction can be realized online without affecting the system response speed during use.

#### 3.3.2. Feature Representation Method Based on One-Dimensional Complex Convolution

Before knowledge transfer, it is necessary to learn as much activity-related experiential knowledge as possible from the data samples in the source domain. In order to effectively mine the time-dimension information of CSI data, a human activity feature representation method based on a one-dimensional complex convolutional network is proposed. The activity features contained in the amplitude and phase of CSI data are extracted by one-dimensional convolution which is suitable for sequence information extraction. Table 1 shows the structure of the feature extraction network model. The 750 × 30 complex CSI matrix is used as the input, and its amplitude and phase are calculated to input the backbone network. The network consists of two one-dimensional complex convolutional layers, two complex batch normalization layers, an adaptive averaging pooling layer, and two complex linear layers. In Table 1, (×4) represents the convolution operation or linear multiplication operation between the real/imaginary parts and the two corresponding network weights four times. (×2) represents two corresponding operations on the real and imaginary parts. Complex convolution operations and complex linear operations are computed in the same way as AP-DCN. The softmax classifier is still used for classification and recognition. Specific network parameters are set as follows: the number of convolution kernels corresponding to the two convolution layers is 128; the kernel size is 3; for one-dimensional convolution, that is, three times the number of input channels. For example, the size of the convolution kernel at the first layer is 3 × 30, and the size of the convolution kernel at the second layer is 3 × 128. The step size and the padding are set to 1.

**Figure 7:**
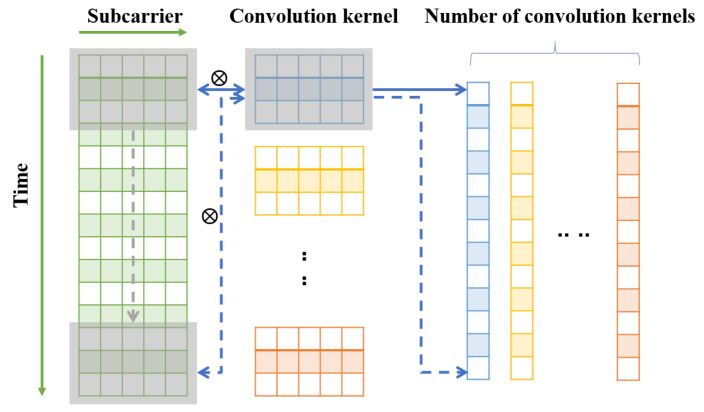
One-dimensional convolution operation.

Figure 7 shows the specific one-dimensional convolution operation process for the real or imaginary part of the complex input matrix. Taking the amplitude or phase of CSI data with an input size of T×s as an example, it is composed of *T* time slices, and each time slice corresponds to *s* subcarrier information, which can be regarded as the feature vector with the dimension of *s*.

The convolution kernel is used to conduct a one-dimensional convolution operation with input data. Different from the two-dimensional convolution operation, the convolution kernel of the one-dimensional convolution operation moves only along the time axis. The convolution kernel is a feature detector, which is equivalent to a sliding time window in the time dimension. We define the number of convolution kernels as *N* and the size of the convolution kernels as *k*. The number of convolution kernels determines the dimension of the output vector, which is the number of features obtained. The size of the convolution kernel determines the time length of the activity involved in each convolution operation. The length of the input data and the size of the convolution kernel determine the number of output neurons. Taking step size 1 and padding size 0 as an example, after a layer of convolution operation, the output matrix with size $N\times \left(T-k+1\right)$ is obtained. For the network mentioned above, the same loss function calculation method, model optimization method, and parameter setting are still used to train the model and obtain the pre-training model for the next stage of knowledge transfer.

#### 3.3.3. Recognition Method Based on Transfer Learning

The above feature representation and learning methods can be used to train the basic model with strong discriminant ability from relatively sufficient source domain data. At this point, the learned knowledge contains the basic characteristics of CSI data and the general characteristics of activities at different source domain locations. When data samples from different locations are unbalanced, to adapt the model to the target domain location where the data sample is further constrained, the model needs to have the ability to transfer knowledge learned from the source domain locations to the target domain locations. Therefore, a multi-location activity recognition scheme based on transfer learning is proposed. The model fine-tuning of transfer learning can realize knowledge sharing with very few target domain samples. The low-level parameters of the network are obtained from sufficient source domain data, and the high-level parameters are learned from the target domain data with limited samples.

**Figure 8:**
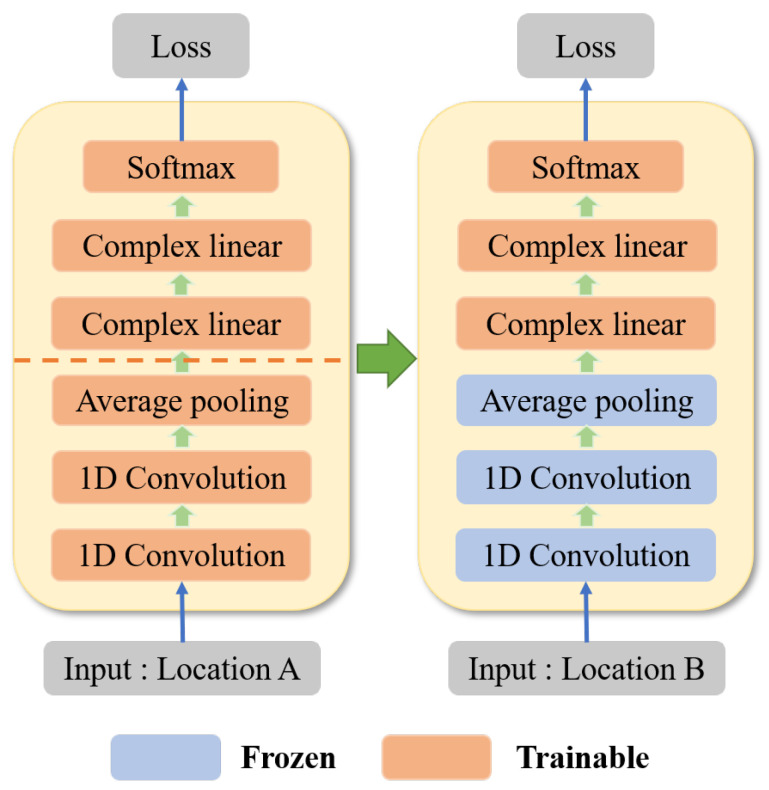
Architecture of transfer learning network.

The transfer learning scheme is based on the pre-training model. Figure 8 shows the architecture of the transfer learning network. The specific process is as follows: Firstly, the network model is pre-trained using the source domain training data set composed of several positions to obtain the optimal model parameters. These parameters are then used to initialize the network and freeze the network layer before the linear layer. Finally, the two linear layers are trained with very few data samples from the target domain locations. Based on the pre-training model, the activity feature representation of source domain learning is transferred to the target domain, which greatly reduces the need for training samples in the target domain and effectively alleviates the problem of sample imbalance. The forward and back-propagation of traditional network training involve all layers of the whole network, while the transfer learning process only involves the last two layers, which can effectively reduce training parameters and shorten training time.

## 4. Experiment and Evaluation

In order to validate the performance of our proposed AP-DCN-based and DCN-TL-based multi-location HAR method, a series of experiments have been conducted. The experiment setup and the experiment results are reported in this section.

### 4.1. Experiment Setup

**Figure 9:**
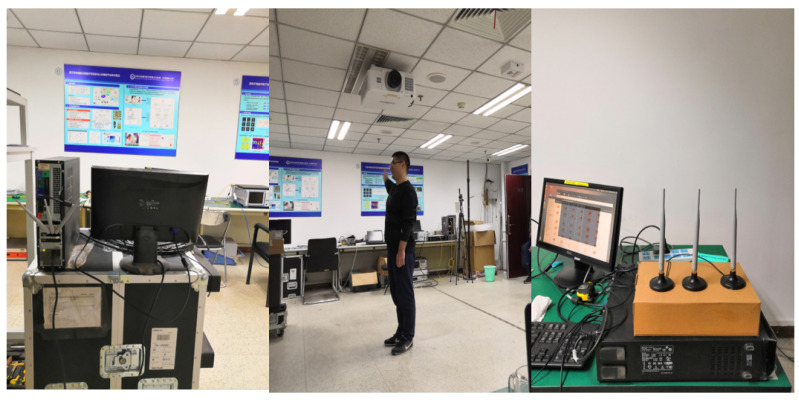
Data collection experimental scene.

To fully evaluate the performance of the proposed method, a dataset has been collected in a cluttered office. The experimental scene is shown in Figure 9. Halperin et al. develop Linux 802.11n CSI Tool [34] based on Intel 5300 Network Interface Card (NIC) which is leveraged to acquire the fine-grained CSI data. The transmitter (TX) and the receiver (RX) work in 802.11n, and operate on a 5 GHz frequency band, with a bandwidth of 20 MHz. They are both equipped with three antennas. CSI with 30 groups of subcarriers from each TX-RX pair can be obtained. It is worth noting that the CSI data from only one of the antenna pairs, namely 30 subcarriers, can be alternatively used.

**Figure 10:**
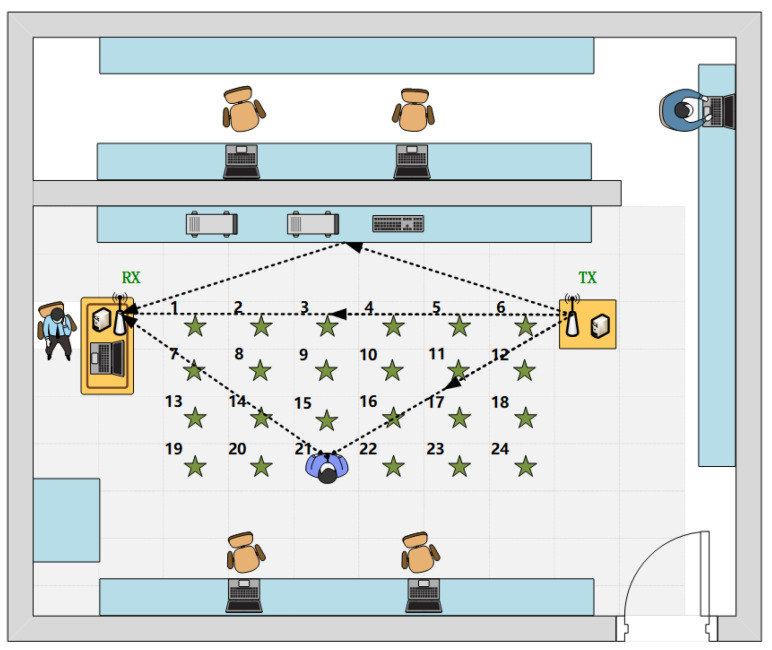
The layout of data collection locations.

To explore the multi-location HAR method, data samples at 24 different locations within a region between the transceivers are collected. The specified location is depicted in Figure 10. The distance between adjacent sampling locations is approximately 0.6 m. The room size is approximately 6 m × 8 m. The distance between TX and RX is 4 m. We predefined five activities, including drawing a circle (O), drawing across (X), lifting up and laying down two arms (UP), pushing and opening with two arms (PO), and sitting down (ST). Five volunteers (one female and four males ranging from 23–30 years old) conducted 50 samples for each activity at each location. There are 24 × 50 = 1200 samples for each activity of each person. Since the initial sampling rate is 200 frames per second, and the actual duration of the actions is 3.5∼4 s, namely 700∼800 frames, 750 frames is cut as a sample.

### 4.2. Experiment Results of Ap-Dcn

The evaluation contains the following three parts. Firstly, the feasibility and effectiveness of the approach are explored. Then, the reliability is discussed. Finally, the proposed method is compared with other approaches to prove the superiority of our system.

**Figure 11:**
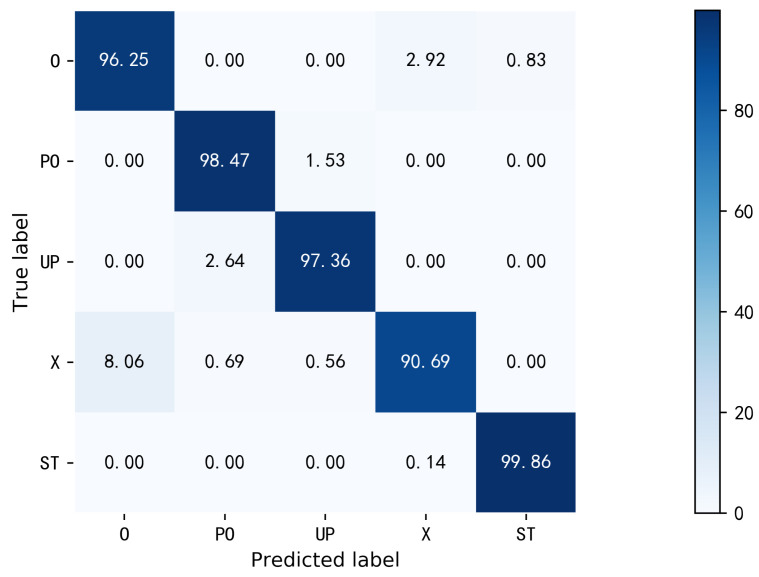
The confusion matrix of AP-DCN recognition accuracy.

**Overall performance.** To verify the feasibility of the multi-location sensing method, 50 samples for each activity at 24 locations of one person are randomly divided into three parts, the training set, the validation set, and the testing set, which accounts for 20%, 20%, and 60%, respectively. It is worth noting that, to reduce the computational burden, only 30 subcarriers from one TX-RX antenna and five training samples from each location are used. The size of the sample is 750 × 30, each is a complex number with its real and imaginary parts. The average accuracy of the proposed method for the five activities of one person is 96.53%. The confusion matrix is demonstrated in Figure 11. It can be seen that all the activities can obtain an acceptable recognition accuracy. In particular, the activity ST achieved 99.86% recognition accuracy. Since X and O are both movements in front of the body after raising the right arm, they are easier to be confused than other activities. In summary, our proposed method performs well in multi-location human activity sensing.

The enhancement effect of amplitude and phase information on multi-location recognition is analyzed. The comparison of recognition accuracy of different methods is demonstrated in Table 2. CNN represents the real-valued convolutional neural network corresponding to AP-DCN network structure. DCN represents complex convolutional networks with the same network structure that are not enhanced by amplitude and phase calculation. Through the comparison between CNN and DCN, it can be seen that complex convolution calculation plays a certain role in extracting richer activity information. The comparison between DCN and AP-DCN shows that manual calculation of amplitude and phase can effectively guide the network to learn more accurate information, so as to achieve higher accuracy of human activity perception.

**Performance of multi-location HAR in terms of different location areas.** According to the principle of wireless sensing, when the target is farther away from the transmitter and the receiver, the delay of the reflected signal generated by the target may be larger. After multi-path superposition, the influence on the received signal is relatively small. Therefore, when the target and the perceptual location area are far from the transceiver, the sensing effect will decrease. To evaluate the reliability of the proposed method in different location areas, four perceptual regions from near and far relative to the transceiver are selected. Table 3 shows the recognition accuracy of different perception areas. Loc1-Loc6 indicates the training and testing samples are selected from location 1–6 in Figure 10. As can be seen, as the location region expands, although the perceptual effect slightly declines, high recognition accuracy can be obtained in each perception area. Although the perception effect is slightly decreased, high recognition accuracy can be obtained in each perception area. For 24 sampling positions covering almost the whole space, the recognition accuracy is still satisfied.

**Performance of multi-location HAR for different number of training samples.** Intuitively, the more samples involved in training, the richer the activity features can be provided. The number of training samples involving 4, 6, 8, and 10 for each activity at each location are investigated. The recognition accuracy with different numbers of training samples is shown in Table 4. As can be seen, the proposed method can provide satisfied recognition accuracy of 95.81% with four training samples. When the number of training samples increases from four to ten, the recognition accuracy is further improved.

**Figure 12:**
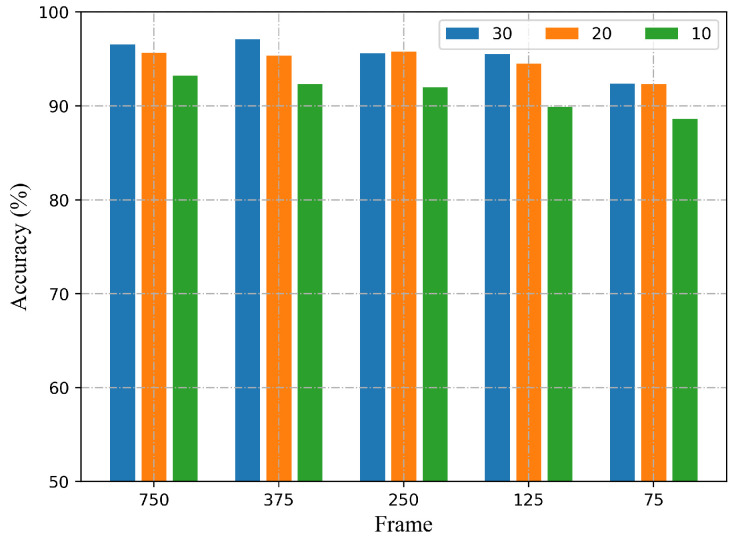
The recognition accuracy with different number of subcarriers and sampling rates.

**Performance of multi-location HAR for different number of subcarriers with different sampling rates.** This paper, in addition to implementing human activity recognition for multi-location, also aims to reduce the computational burden, which is more suitable for real-time applications. Therefore, a small sample size is desired. CSI measurements are collected at the initial transmission rate of 200 packets per second, and the 750 CSI series are down-sampled to 375, 250, 150, and 75. Furthermore, the number of subcarriers of 10, 20, and 30 are investigated. It is worth noting that only five training samples for each activity at each location are utilized. The recognition accuracy with different numbers of subcarriers and sampling rates are shown in Figure 12. As can be seen, the proposed method can provide satisfied recognition accuracy with very few subcarriers and low sampling rates. As far as the sampling rate is concerned, when the sampling rate decreases to 20 frames/s, the method can still obtain 88.61% with only 10 subcarriers.

**Performance of multi-location HAR for different persons.** To verify the reliability of the system for different users, we collected the data samples involving five subjects marked as User1–User5. Their heights range from 160–180 cm, while the age is from 23–30 years old. The recognition results of the five users for five activities at 24 locations are shown in Table 5. As illustrated, the average recognition accuracy is 96.85%. Consequently, our method can work well for different users.

**Comparison with different recognition methods.** In this part, to evaluate the superiority, four typical approaches are compared with our system. ActNet [26] is the state-of-the-art multi-location HAR method, which decomposes the input samples into the location-irrelevant activity features and activity-irrelevant location features. It jointly learns different activities from multi-locations to mitigate the issue of insufficient data. SqueezeNet [35] and Alexnet [36] are two classical deep learning methods. WiHand [37] utilizes the low rank and sparse decomposition (LRSD) algorithm to separate activity signal from background information, thus making it adapt to location variation. It is worth noting that, in order to keep the settings as similar as possible to the original literature, all five methods use 10 training samples. In addition, the first three use 270 subcarriers, while the last two use 30 subcarriers. As can be seen in Table 6, our system outperforms these three methods in multi-location HAR, even using fewer subcarriers.

### 4.3. Experiment Results of Dcn-Tl

This section still uses the data set composed of five human activities collected at 24 positions in Section 4.1 to evaluate the performance of the multi-location human activity recognition method based on DCN-TL. 50 samples of each activity collected by volunteers at each location are divided into three parts: model training, knowledge transfer, and performance test, accounting for 60%, 20%, and 20%, respectively. For any volunteer, a maximum of 30 training samples, 10 transfer samples, and 10 test samples are available for an activity at each location. This section still uses 750 frame length and 30 subcarriers as input. The parameters of the pre-training network are the same as in the previous section.

**Figure 13:**
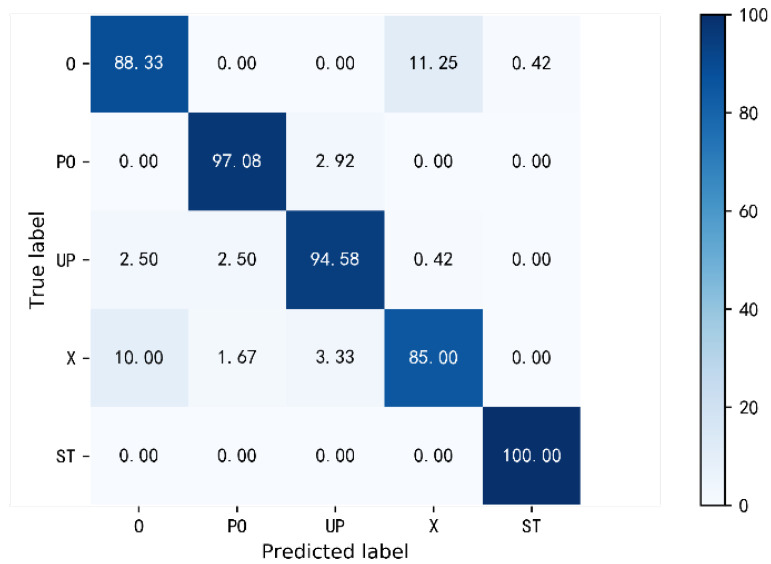
The confusion matrix of DCN-TL recognition accuracy.

**Overall performance.** In order to verify the perceptual performance of the method when the number of training samples at different locations is unbalanced and the number of samples at some locations is further limited, for the 24 sampling locations in the data set, we take the example of sufficient samples at six locations and insufficient samples at other locations to evaluate the feasibility of the method. The six training positions are selected starting from the first position in Figure 10 at equal intervals, taking one for every four positions from location 1 to 24. Three samples were randomly selected from 10 transfer samples for model transfer learning. The testing set consists of testing samples involving five activities at 24 positions, with a total of 24 × 5 × 10 = 1200 samples. Experimental results show that the average recognition accuracy of DCN-TL is 93.00%. The confusion matrix is shown in Figure 13. Among them, ST can obtain 100% recognition accuracy. Other activities can also obtain satisfactory recognition accuracy. Therefore, DCN-TL performs well in the multi-location human activity sensing when the number of training samples at different positions is unbalanced and the number of samples at some positions is further limited.

**Performance of multi-location HAR in terms of different location areas.** We discuss the performance of human activity recognition based on the DCN-TL recognition method when the perception area gradually expands. At the 24 sampling positions shown in Figure 10, location 1–6 in the first row parallel to the transceiver is defined as perception area 1, and the experiment number is marked as N1. Training positions 2 and 5 are symmetrically selected. One row is added at a time to gradually expand the perception area, forming evaluation experiments numbered N2, N3, and N4. The training position of the latter perception area is increased based on the training position of the former perception area. For example, “N1+8/11” represents that the training positions 8 and 11 are added on the basis of N1, namely, the four positions participating in the model pre-training are 2/5/8/11. Table 7 shows the recognition accuracy of different perception areas. It can be seen that, with the expansion of the perception area, the recognition accuracy gradually improves. This is because the model can learn more knowledge in the pre-training stage due to the gradual increase of training positions.

**Performance of multi-location HAR in terms of different number of training locations.** The number of positions involved in pre-training is a critical factor affecting perceptual performance. The influence of the number of locations involved in pre-training on recognition accuracy is discussed in this part. A total of 12, 8, 6, and 4 pre-training positions are sampled at equal intervals from 24 positions. As shown in Table 8, when only four positions participate in training, and three samples are provided for each transfer location, the recognition accuracy can still be 90.42%. When there are 12 training positions, the recognition accuracy is 94.64%. With the increase of the number of training positions, the recognition accuracy increases gradually.

**Performance of multi-location HAR in terms of different number of transfer samples.** The influence of the number of samples involved in knowledge transfer on recognition accuracy is discussed. This part takes six pre-training positions as examples and tests the method at 24 positions. A total of 1–5 transfer samples are randomly selected from the transfer sample set. The recognition accuracy is shown in Table 9. When only one transfer sample is provided, the recognition accuracy is 90.55%. Using five transfer samples, the recognition accuracy can reach 97.44%. With the increase in the number of transfer samples, the model can learn more activity characteristics of the target domain location based on the experiential knowledge of source domain location learning, so as to improve the recognition accuracy.

**Performance of multi-location HAR for different users.** To verify the reliability of the proposed method for different users, the performance of human activity data involving five volunteers are evaluated. Five volunteers are marked as User 1–User 5. Six positions are used for training, 24 positions are tested, and three transfer samples are provided for each position. Table 10 shows the recognition accuracy of 5 activities at 24 positions by different volunteers. The average recognition accuracy is 94.02%. Experimental results show that this method can be well applied to different users.

## 5. Discussion

In the evaluation for this paper, 24 positions with an interval of 0.6m are sampled in a typical indoor area. When the range of sensing area is fixed, increasing the number of sampling locations will improve the perception effect. If the sensing area continues to expand, such as in a larger room, the perception effect will be decreased to some extent. When the sensing target is far away from the transmitter and receiver, the influence on signal transmission will be weakened. Theoretically speaking, the sensing performance decreases with the increase of the distance between the sensing target and the sensing device. For the number of activities, there may be more activities in practice scenarios. As for the experimental settings in most literature, five to eight activities are usually recognized in a typical smart home control scenario. If the number of activities continues to increase, the recognition accuracy will decrease to a certain extent, because some actions may have similar features and be easily confused. This is still a challenging issue for Wi-Fi-based human activity recognition, which will be further explored in future work.

## 6. Conclusions

In this paper, Wi-Fi-based multi-location human activity recognition technique is explored. A novel AP-DCN-based method that fully leverages the amplitude and phase information is presented. The complex convolution layer, complex batch normalization layer, and complex ReLU activation function are leveraged for feature representation. Furthermore, considering the unbalanced sample number at different locations, a perception method based on DCN-TL is proposed. To verify the performance of the method, a dataset involving five activities at 24 positions in an office is built. The experiment results indicate that the AP-DCN-based method can achieve an average accuracy of 96.85% for five people with only five training samples at each of the 24 locations. Furthermore, the proposed method is also applicable to the training samples with a low sampling rate and fewer subcarriers. In the case of unbalanced number of data samples at different locations, the recognition accuracy is 94.02%. Therefore, it is concluded that the presented method is feasible for multi-location human activity recognition with limited data samples, which promisingly promotes the generalization performance of the device-free sensing system.

## Figures and Tables

**Table 1:** The model structure of feature extraction network.

Layer	Output Size
Input layer	(−1, 30, 750)
Complex convolution layer 1 (×4)	(−1, 128, 750)
Complex batch normalization layer 1 (×2)	(−1, 128, 750)
Complex convolution layer 2 (×4)	(−1, 128, 750)
Complex batch normalization layer 2 (×2)	(−1, 128, 750)
Adaptive average pooling layer (×2)	(−1, 128)
Complex linear layer 1 (×4)	(−1, 64)
Complex linear layer 2 (×4)	(−1, 5)

**Table 2:** Comparison of recognition accuracy of different methods.

Sensing Method	Accuracy (%)
CNN	92.23
DCN	94.92
AP-DCN	96.53

**Table 3:** The AP-DCN recognition accuracy of different sensing areas.

Sensing Area	Accuracy (%)
Loc 1∼Loc 6	98.44
Loc 1∼Loc 12	98.27
Loc 1∼Loc 18	97.99
Loc 1∼Loc 24	96.53

**Table 4:** The recognition accuracy for different number of training samples.

Number of Training Samples	4	6	8	10
Accuracy (%)	95.81	96.85	97.39	98.11

**Table 5:** The AP-DCN recognition accuracy for different users.

Users	User1	User2	User3	User4	User5	Average
Accuracy (%)	96.53	98.00	96.28	95.00	98.42	96.85

**Table 6:** Comparison with different recognition methods.

Methods	Accuracy (%)
ActNet [26]	94.60
SqueezeNet [35]	90.07
Alexnet [36]	89.00
WiHand [37]	88.22
AP-DCN	98.11

**Table 7:** The DCN-TL recognition accuracy of different sensing areas.

Number	Training Locations	Testing Locations	Accuracy (%)
N1	2/5	Loc 1∼Loc 6	88.33
N2	N1+8/11	Loc 1∼Loc 12	90.83
N3	N2+14/17	Loc 1∼Loc 18	91.78
N4	N3+20/23	Loc 1∼Loc 24	93.42

**Table 8:** The recognition accuracy of different numbers of pre-trained locations.

Number of Training Locations	24	12	8	6	4
Accuracy (%)	98.58	94.64	93.33	93.00	90.42

**Table 9:** The recognition accuracy of different numbers of transfer samples.

Number of Transfer Samples	1	2	3	4	5
Accuracy (%)	90.55	93.17	94.75	96.33	97.44

**Table 10:** The DCN-TL recognition accuracy for different users.

Users	User1	User2	User3	User4	User5	Average
Accuracy (%)	93.50	93.00	94.75	93.67	95.17	94.02

## Data Availability

Not applicable.
